# Masters of adaptation: How cancer and immune cell plasticity mediates tumor progression

**DOI:** 10.1371/journal.pbio.3003301

**Published:** 2025-07-15

**Authors:** Sheera R. Rosenbaum, Kaiah M. Fields, Heide L. Ford

**Affiliations:** 1 Department of Pharmacology, University of Colorado Anschutz Medical Campus (AMC), Aurora, Colorado, United States of America; 2 Molecular Biology Program, University of Colorado Anschutz Medical Campus (AMC), Aurora, Colorado, United States of America; Princeton University, UNITED STATES OF AMERICA

## Abstract

One of the greatest challenges to cancer therapy is tumor cell plasticity. Cancer cells can rapidly alter their phenotype to promote survival and evade immune cell attack, while the plasticity of other cells in the tumor microenvironment (such as immune cells, which need to be able to respond to a diverse range of bodily threats) can be leveraged to further promote tumor growth and progression. This Essay discusses the mutual plasticity of cancer and immune cells, with a focus on epithelial–mesenchymal plasticity in tumor cells, and explores how this interplay contributes to tumor progression and can be targeted therapeutically.

## Abstract 2

The ability of cancer cells and immune cells cell to dynamically change their phenotype contributes to tumor progression and therapy resistance. This Essay explores the mutual plasticity of various cell types in the tumor microenvironment and discusses implications for the development of therapeutic strategies.

## Introduction

**Fig 1:**
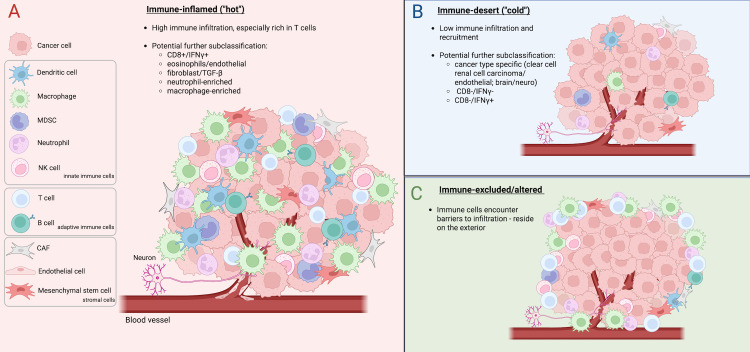
The tumor immune microenvironment. Key cell types within the innate immune system, adaptive immune system and tumor stroma, as well as the tumor vasculature and innervation, are portrayed in this rendering of the tumor immune microenvironment (TiME). **A.** Immune-inflamed TiME, replete with various immune cell subtypes. **B.** Immune-desert TiME, lacking proper immune recruitment. **C.** Immune-excluded/altered TiME, recruiting immune cells but preventing their infiltration. Created using BioRender, https://BioRender.com/mbxvkmp. CAF, cancer-associated fibroblast.

Cancer is a complex disease that can be driven by, but also eliminated through, its continuous communication with the immune system. The immune surveillance hypothesis proposes that the immune system can detect and eliminate emerging tumors, but that tumors evade this surveillance through complex crosstalk with immune cells that results in tumor cell escape from the anti-tumor immune response [1]. During tumor development, selection pressures shape the cellular composition of the tumor immune microenvironment (TiME), giving rise to distinct tumor immune subtypes which can be broadly categorized as: immune-inflamed (also known as ‘hot’; replete with anti-tumor T cells); immune-desert (also known as ‘cold’; lacking an active immune response); and immune-excluded/altered (immune cells are prevented from infiltrating) [2] (Fig 1). Multiple groups have defined immune hot/cold tumor subtypes more precisely, characterizing these subtypes by enrichment of specific immune cells and immune cell activity [3–5], as well as by distinct patterns of gene expression [2,3] or by being specific to certain cancer types. Importantly, these categories are associated with varied responses to immunotherapy, with immune-inflamed and T-cell-enriched tumors showing the best responses [2]. Thus, identifying the mechanisms that shape the TiME will be critical to inform immunotherapeutic strategies.

Tumor heterogeneity and tumor cell plasticity contribute considerably to the tumor–immune crosstalk that promotes the development and maintenance of the TiME. Cancer cell plasticity can change the immune cell composition of tumors and the effectiveness of anti-tumor immune responses. In response to tumor cell signaling, immune cells also adapt their phenotype and behavior, which can contribute positively or negatively to anti-tumor immune responses. This bidirectional plasticity (the ability of cancer cells to shape immune phenotypes and vice versa) has profound implications for therapy. In this Essay, we explore how immune cells promote cancer cell plasticity and how cancer cells can reciprocally promote plasticity in immune cells, focusing on epithelial-to-mesenchymal transition (EMT) as a major form of cancer cell plasticity, but also examining other forms of tumor cell plasticity.

## Tumor heterogeneity and plasticity affect the TiME

### Tumor heterogeneity

Tumor heterogeneity, referring to differences in cancer cell composition within individual tumors or between different patients, can be attributed to a variety of factors including genetic alterations, epigenetic shifts, microenvironmental cues and dynamic changes in differentiation states [6,7]. Heterogeneous cancer cells communicate with one other to cooperate and survive immune attack, leading to enhanced tumor growth [8,9]. Intra-tumoral heterogeneity is associated with decreased immune cell infiltration and response to immunotherapy [10], and higher heterogeneity scores are associated with reduced overall survival in patient datasets across multiple tumor types [10–12]. While clonal cooperation can promote resistance to T cell responses [8], heterogenous loss of MHC class I in spatially distinct subclonal lesions is accompanied by an increase in natural killer (NK) cell infiltration [9]. This change in immune infiltration is significant, as many immunotherapies approved by the US food and drug administration (FDA) focus on boosting T cell activity, when a combination of therapies that boost both T cell and NK cell activities is likely to be more efficacious [9]. Thus, while tumor heterogeneity often poses a clinical barrier to treatment, such heterogeneity can also be exploited for the development of novel therapeutic strategies.

In addition, immune and stromal cell interactions promote adaptive changes that contribute to tumor heterogeneity [13], an effect that is impacted by the spatial distribution of cell types within the TiME. For example, immune cells is the TiME locally secrete different cytokines that then alter gene expression in a spatially distinct manner, creating heterogeneous phenotypes of cancer cells across the tumor [14]. In this way, the spatial confinement of cellular crosstalk creates an additional source of intra-tumor diversity. Capturing the complexity of heterogeneous interactions in model systems (Box 1), especially with respect to the spatial and temporal components of these interactions, poses a challenge. Thus, tumor heterogeneity is influenced by dynamic interactions within the TiME and has important implications for anti-tumor immune responses.

**Box 1 Model systems for studying dynamic changes in the tumor immune microenvironment.**

**Organoids**

Tumor organoids are *in vitro* models that recapitulate the 3D structure of the tumor [15]. Organoids can consist of cancer cells alone or can incorporate other cell types from the tumor immune microenvironment (TiME) [16], and growth conditions can be optimized to maintain tumor cell heterogeneity *in vitro*. Patient-derived xenografts can also be used for organoid generation, and multiple groups have developed patient-derived xenograft organoid models as a platform to screen compounds for personalized patient treatment [17]. Importantly, differences have been observed in the efficacy of therapeutic interventions in 2D models compared to 3D organoids or *in vivo* models [18], emphasizing the importance of modeling complex 3D cellular interactions *in vitro*.

**Mouse models**

Mouse models allow researchers to study tumor–immune interactions and how they impact tumor growth *in vivo* under controlled conditions. Syngeneic tumor models are models in which mouse-derived tumor cell lines are injected into immune competent mice of the same genetic background [19] (e.g., E0771 breast cancer cells and C57BL/6 mice). Genetically engineered mouse models are strains of mice that have been modified to spontaneously develop tumors, and can be either overexpression (OE) or knockout (KO) models [20]. Both OE and KO mice can have either a single gene altered in the entire mouse, or be designed to alter the gene in a single organ or cell type within the mouse (thus conditional KO or OE). The aforementioned models enable investigators to examine tumors in the context of an intact immune system, but are limited by differences between the human and mouse immune systems. To better understand the interaction between human immune and human cancer cells, humanized mice have been developed in which human immune cells are engrafted into immunodeficient mice (e.g., NSG mice) [21]. However, humanized mice do not completely recapitulate the human immune system. For example, most models lack secondary lymphoid structures that are critical for the maturation of certain immune populations. These mice also lack human cytokines that would otherwise aid engraftment of human immune progenitors. Furthermore, there are batch effects between humanized mice and limitations to the available human tissue used for their generation [22]. In sum, mouse models are central to the study of cancer immunology, and there is consistent effort towards improving current models and developing new models.

**Lineage tracing models**

Tumor heterogeneity and cell plasticity play key roles in regulating cellular interactions in the TiME, and various tools have been developed to investigate changes in tumor composition that contribute to TiME crosstalk. Phenotypic shifts, such as epithelial-to-mesenchymal transition (EMT), can be monitored during tumor progression through the use of lineage tracing models, such as those that follow N-cadherin expression [23] or follow unique barcodes genetically encoded into individual tumor cells [24–26]. Barcoding has yielded additional insights into specific cancer cell subclones that promote metastasis [25,27] and resistance to therapy [28–30]. Newer technologies have coupled cell barcoding with multiomic analyses for higher resolution of dynamic changes to subclonal cancer cell populations [31,32]. Labeling and tracing techniques have also been applied to immune cells to follow changes in immune cell infiltration over time [33] and the dynamics of T cell [34] and macrophage [35] responses in the tumor.

### Tumor cell plasticity

Tumor cell plasticity refers to adaptive tumor cell changes in response to various cues, which contributes to both the generation and maintenance of tumor heterogeneity by dynamically introducing diverse features to individual cancer cells within a given tumor. This plasticity can be attributed to multiple influences including transcription factor reprogramming, stemness, microenvironmental crosstalk, extracellular matrix interaction, adaptive resistance to therapy and metabolic pressure, among others [36]. In this Essay, we focus on one extensively studied mechanism of plasticity, EMT, as a critical means by which the TiME is altered, promoting crosstalk between tumor and immune cells.

EMT is an evolutionary conserved developmental program (Box 2) usurped by carcinoma cells to shift from an epithelial, proliferative state to a mesenchymal-like, invasive state during tumor progression to promote cancer cell invasion and metastasis [37]. This transition can be induced through multiple mechanisms, including activation of EMT transcription factors, exposure to cytokines such as TGF-β, and signaling downstream of tyrosine kinase receptors [38–40]. Importantly, both the dynamic process of immunoediting (Box 2) [41] and inflammation [42] promote tumor cell EMT, and many factors that induce EMT can also influence immune cell function [43], highlighting a critical role for immune–cancer crosstalk in shaping cancer cell phenotypes. Cells may undergo a partial EMT, exhibiting various EMT-like features along a spectrum with the ability to adopt mixed features, also known as epithelial–mesenchymal plasticity, and a single tumor may contain a mix of cells in different hybrid EMT states [39]. In heterogeneous tumors, the interaction between cell types in various states of EMT can have important consequences, and cells that have undergone EMT can enhance the EMT-like characteristics and invasiveness of neighboring cells [44,45]. Furthermore, cells in a hybrid EMT state or tumors with cells in mixed EMT states can protect against immune attack and surveillance to promote progression [46–48]. Thus, the increased heterogeneity resulting from epithelial–mesenchymal plasticity can contribute to tumor progression and aggressiveness.

**Box 2 Glossary.**

**Aerobic glycolysis**

A metabolic process in which cells convert glucose to lactate even in the presence of oxygen (called the Warburg effect when occurring in cancer cells).

**Cytotoxic killing/cytolysis**

The process by which cytotoxic immune cells, such as CD8^+^ T cells and NK cells, kill infected or cancerous cells.

**Developmental program**

A set of tightly controlled biological instructions that guide cells through specific stages of growth, specialization and function during an organism’s formation, which can also be reactivated in cancer.

**Extravasation**

The process by which cancer cells move from blood vessels into surrounding tissue; a critical step of metastasis.

**Immunoediting**

The dynamic process by which the immune system interacts with developing cancer cells, shaping their evolution over time.

**Macrophage polarization**

The process by which macrophages adopt different functional states in response to environmental signals.

**‘Missing-self’ recognition**

When cells have lost or have reduced expression of MHC class I molecules on their cell surface, NK cells recognize those cells as foreign and are activated to eliminate them.

**Necroptosis**

A pro-inflammatory form of programmed cell death.

**Neoantigen**

A tumor-specific antigen that results from cellular transformation and is therefore not produced by healthy tissue.

Cancer cell EMT is associated with changes in gene expression that exert wide-ranging effects on immune cells in the TiME. Recent work has expanded on EMT gene expression changes, demonstrating that two distinct EMT programs arise to drive either an EMT-invasive program or an EMT-inflammatory program [49], and that tumors may possess nonoverlapping subpopulations that have activated these two programs. Importantly, the EMT-inflammatory program is associated with multiple changes in cytokines, which can affect immune cell trafficking and activity, and increased infiltration of tumor-associated macrophages [49]. This finding is in line with previous studies demonstrating that EMT induction is associated with increased expression of cytokines that promote infiltration of tumor-associated macrophages and myeloid-derived suppressor cells (MDSCs) [50], both of which promote tumor growth.

Beyond myeloid cells, crosstalk between cancer cells that have undergone EMT and immune cells can induce CD4^+^ T cell differentiation to an immunosuppressive T regulatory cell subtype [51], inhibit proliferation and infiltration of B cells, NK cells and CD4^+^ and CD8^+^ T cells [51,52], and render cancer cells less sensitive to cytotoxic T cell killing (Box 2) [53]. While EMT suppresses T cell killing, it may have the opposite effect on NK cells, where it upregulates NK cell activating ligands, rendering the cancer cells more sensitive to NK cell cytolysis [54]. EMT can also reduce cell adhesion, leading to the formation of monoclonal cell clusters that are more susceptible to NK cell elimination and less effective at colonizing metastatic sites compared to polyclonal cell clusters [55]. Thus, rather than T-cell-based immunotherapies, therapeutic strategies that consider NK cell responses may be more suitable for heterogeneous tumors in which some of the cells have undergone EMT.

## Immune induction of tumor cell plasticity

**Fig 2:**
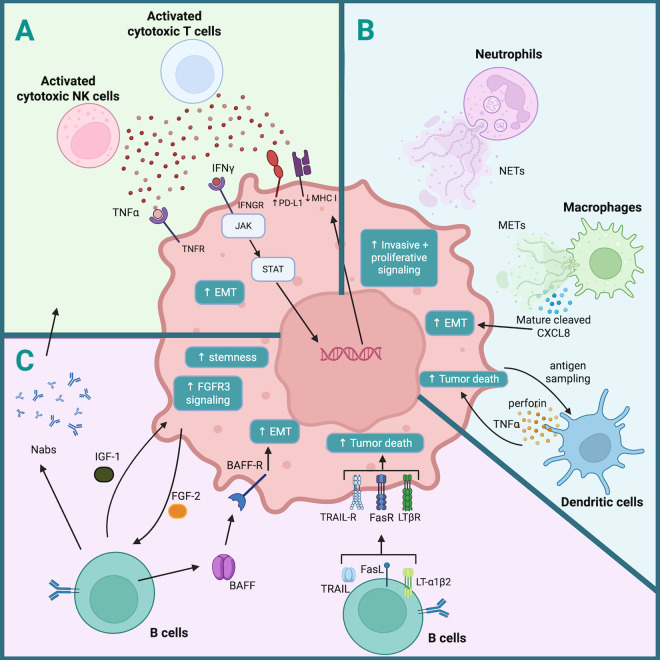
Immune induction of tumor cell plasticity. Shown is an overview of mechanisms by which immune cells alter tumor cell phenotype and activity. A. Cytotoxic cell induction of dynamic tumor changes. B. Myeloid cell modulation of tumor cells. C. The role of B cells in shaping tumor cell plasticity and the TiME. Created using BioRender, https://BioRender.com/d6ler25. EMT, epithelial-to-mesenchymal transition; METs, macrophage extracellular traps; Nabs, natural antibodies; NETs, neutrophil extracellular traps.

Although tumors are inherently heterogeneous, interactions with immune cells can cause cancer cells to undergo dynamic changes that further intensify this heterogeneity, including induction of EMT. In this section, we highlight immune–tumor interactions that elicit phenotypic changes in cancer cells (Fig 2).

### Cytotoxic cell induction of dynamic tumor changes

Cytotoxic killing of cancer cells is largely executed by cytotoxic CD8^+^ T cells, effectors of the adaptive immune system, and NK cells, effectors of the innate immune system. Although CD8^+^ T cells are activated in an antigen-specific manner whereas NK cell activation is antigen-independent, once activated, both of these cell types mediate cytotoxic killing of target cells through similar mechanisms [56,57]. Tumor cells undergo substantial changes under such cytotoxic immune cell pressure (Fig 2A). CD8^+^ T cell engagement of cancer cell surface MHC–antigen complexes leads to T cell activation; therefore, loss of antigen expression or presentation on cancer cells is a common adaptation to escape from T cell recognition [58]. However, loss of MHC class I cell surface expression promotes NK cell ‘missing-self’ recognition (Box 2) and subsequent activation, rendering cancer cells that have undergone antigen escape more vulnerable to NK cell elimination [59]. In response to IFNγ release by CD8^+^ T cells and NK cells, tumor cells activate JAK–STAT signaling and up-regulate immunosuppressive proteins, such as PD-L1 and IDO [60,61] (Fig 2A), which support cancer cell resistance to cytotoxic killing by both T cells and NK cells [60–62]. Cytotoxic T-cell-secreted IFNγ also contributes to cancer cell immunoediting by inducing copy number rearrangements that directly lead to loss of antigen and immune escape [63]. TNFα secretion by cytotoxic cells can also directly promote cancer cell dedifferentiation to an immune resistant phenotype and promote antigen downregulation [64].

Cancer cell phenotype switching has been commonly observed under cytotoxic pressure. Breast cancer cells undergo EMT in response to CD8^+^ T cells, reverting to a stem cell-like phenotype [65]. Similarly, in co-cultures of NK cells and melanoma cells, melanoma cells phenotypically shifted from an epithelial/proliferative state to a more mesenchymal-like/invasive state [66]. In lung adenocarcinoma, tumor cell subpopulations adopted developmental states that fell along a continuum of developmental progression [67], but pressure from NK cell immunosurveillance eliminated those cells in a regenerative state (marked by high SOX2), instead allowing for outgrowth of metastasis-initiating cells (marked by high SOX9). This study highlights the role of NK cells in influencing developmental plasticity during metastatic progression. While cancer cells undergo dynamic alterations in response to cytotoxic immune stimulation, tumor immunoediting also creates an evolutionary bottleneck that allows for resistant subclones that are initially present in the tumor to grow out [68]. Thus, both heterogeneity and phenotypic plasticity critically contribute to the ability of tumors to adapt to and survive cytotoxic attack.

### Myeloid cell modulation of tumor cells

Myeloid cells, which include macrophages, dendritic cells (DCs), neutrophils and MDSCs, amongst other cell types, are part of the innate immune system [69] and are not only plastic themselves, but promote plasticity amongst tumor cells [70]. Accordingly, they are interesting cellular populations to target therapeutically, as it may be possible to boost their anti-tumor functions or to inhibit their pro-tumor functions.

Neutrophils are typically short-lived and rapidly recruited from the bloodstream in response to infection, injury or cancer [71]. Like many myeloid cells, neutrophils can have pro-tumor or anti-tumor effects depending on their environment [72]. Neutrophils can promote breast tumor aggressiveness in an MMTV-PyMT mouse model by engaging in direct physical interactions with tumor cells, as well as through indirect interactions, that together promote tumor growth, angiogenesis and metastasis by stimulating invasive and proliferative signaling programs within cancer cells [73]. A prominent characteristic of neutrophils is their ability to form neutrophil extracellular traps (NETs), which participate in pathogen response, but also encourage a pro-metastatic environment in the context of chronic stress (Fig 2B) [74].

A similar, yet less well-known process that also alters tumor function is the release of macrophage extracellular traps (METs). Macrophages are best known for their ability to phagocytose diseased cells, cellular debris or pathogens, but are also critical within the TiME as communicators between different cells types [75]. Chen and colleagues discovered crosstalk that occurs between macrophages and pancreatic cancer cells in which cancer cell necroptosis (Box 2) enhances a phagocytosis resistance signal, which in turn induces MET release that promotes liver metastasis [76]. METs, like NETs, are web-like structures consisting of filaments that contain DNA and proteins that are released from cells, often during a unique form of cell death. METs serve multiple purposes; in this case, acting as a scaffold for the cleavage of tumor-secreted CXCL8 into its active, pro-metastatic form (Fig 2B) [76]. Through CXCL8 signaling and other mechanisms, METs influence tumor cells and their ability to metastasize by initiating EMT, degrading the extracellular matrix and adhering to endothelial cells during extravasation (Box 2). Others have reported that macrophages additionally act on tumor cell plasticity at metastatic sites by secreting IL-35, which activates the JAK2–STAT6–GATA3 signaling axis in cancer, thus facilitating EMT reversal and tumor colonization at these secondary sites [77].

Another myeloid population that influences tumor progression is DCs, professional antigen presenting cells that stimulate naïve T cells and are key orchestrators of anti-tumor immunity [78]. While most anti-tumor effects of DCs occur through their activation of cytotoxic cells, DCs can also directly signal to tumor cells and induce cell death through TRAIL, nitric oxide, perforin, granzyme B and TNFα (Fig 2B) [79–81]. For example, DC-secreted TNFα can promote RIPK-dependent apoptosis in tumor cells, which allows for sampling of tumor antigen by DCs and further induction of anti-tumor immunity [80]. Together, these examples underscore the influence of myeloid cells on tumor plasticity and progression, highlighting their dual roles as both modulators of tumor behavior and potential therapeutic targets across diverse cancer contexts.

### B cells shape tumor cell plasticity

Recent studies have uncovered the importance of B cells, an adaptive immune population best known for its role in producing antibodies, to the tumor immune response. There are several types of B cells, including plasma cells, memory B cells and regulatory B cells, with subsets existing within each category that arise from pressures including crosstalk with the TiME and spatial localization within the tumor [82]. B cells have emerged as a new target for cancer immunotherapy, as more than half of existing studies indicate a positive prognostic role for intra-tumoral B cells, and their heterogeneity can be exploited therapeutically [83]. Rawat and colleagues challenged the idea that B cells play an insignificant role in tumor immunity by questioning the use and utility of the most commonly used B cell deficient mouse—μMT [84]. They determined that B cells and the natural antibodies (Nabs) they produce are critical to the detection and elimination of neoantigen-expressing cells (Box 2), such as cancer cells (Fig 2C) [84]. In addition to the importance of Nabs to anti-tumor immunity, B cells play a role in T cell activation through antigen presentation [85,86]. In a tumor context, these B cell–T cell interactions often occur within tertiary lymphoid structures [87], ectopic lymphoid organs that develop in tumors and other sites of chronic inflammation and may be predictive of prognosis and therapy response [87]. Furthermore, B cells themselves can act as cytotoxic cells and directly kill cancer cells. For example, B cells can act in a cytotoxic manner against tumor cells in leukemia and breast cancer through their expression of Fas ligand and other TNF superfamily members such as lymphotoxin and TRAIL [88,89].

While many functions of B cells support tumor clearance, B cells can also regulate tumor cell plasticity and heterogeneity in a pro-metastatic manner. In pancreatic cancer, B cells secrete high levels of BAFF, which induces an EMT gene signature in BAFF-receptor-expressing pancreatic cancers [90]. In bladder cancer, B cells increase invasion and metastasis through their expression of IL-8, which promotes increased matrix metalloproteinase secretion and invasive potential in cancer cells via androgen receptor signaling [91]. Co-culture of B cells and melanoma cells leads to reciprocal changes in both cells, whereby tumor cell secretion of FGF2 induces B cell increases in FGFR1/FGFR3 signaling and IGF1, IL-1α/β and PDGF-A/B expression. B cell secretion of IGF1 then causes tumor cells to activate FGFR3 signaling and adopt stem-like properties (Fig 2C) [92]. This study further demonstrates that the induction of stem-like subpopulations, provoked by tumor-associated B cells, contributes to enhanced tumor heterogeneity and resistance to targeted therapy. As modulators of both anti-tumor immunity and tumor cell plasticity, B cells represent a promising yet complex target for therapeutic intervention within the TiME.

## Tumor induction of immune cell plasticity

Immune cells are highly responsive to environmental cues and rely on instruction from multiple cell types that cooperate to mount an effective response against infections or, in the case of cancer, against abnormal cells in the body. While this plasticity is critical for productive anti-tumor immunity, it can also be manipulated by cancer cells to promote tumorigenic growth, some examples of which are discussed below and summarized in Table 1.

**Table 1:** Examples of immune cell reprogramming resulting from crosstalk with cells in the tumor immune microenvironment.

Immune cell	Interacting cell	Examples of how cellular crosstalk affects immune cells	References
Macrophages	Cholangiocarcinoma cells	Suppression of macrophage antigen presentation via increased α-ketoglutarate production.	[93]
	Breast cancer cells	Upregulated CECR2 in cancer cells increases CSF1, CSF2, CSF3 and CXCL1 to promote macrophage polarization.	[94]
	Tumor cells (multiple cancer types)	Lactate accumulation as a result of cancer cell aerobic glycolysis and fatty acid metabolism induces macrophage polarization.	[95–98]
	Neutrophils	Reprogramming to pro-tumor phenotype.	[99]
	Endothelial cells	Polarize to an immune-suppressive phenotype.	[100]
Macrophages and monocytes	CAFs	Polarize to an immune-suppressive phenotype.	[101,102]
MDSCs	Tumor cells (multiple cancer types)	Expression of epigenetic regulators in tumor cells leads to increased recruitment of MDSCs.	[103–106]
Neutrophils	Tumor cells (multiple cancer types)	Cancer cell production of lactate increases neutrophil release of pro-tumor factors and NETs.	[107,108]
	Pancreatic cancer cells	Cancer cell expression of methyltransferase SETD2 promotes neutrophil recruitment.	[109]
	Colorectal cancer cells	Adoption of pro-tumor phenotype after uptake of cancer cell apoptotic debris.	[99]
DCs	Melanoma cells	Impaired differentiation, activation and function.	[110]
	Tumor cells (multiple cancer types)	Increased accumulation of lactate and lipids impairs antigen presentation.	[111,112]
CD8^ +^ T cells	Breast cancer cells	Suppression of T cell anti-tumor immune response via expression of PD-L1.	[113]
T cells	Tumor cells (multiple cancer types)	Metabolic abnormalities and impaired function caused by mitochondrial dysfunction, as a result of hypoxia induction or transfer of mutant mitochondrial DNA.	[114,115]
	CAFs	Support anti-tumor T cell responses in gastric cancer.	[116]
	Osteoclasts	Suppress T cell cytotoxic killing.	[117]
NK cells	Tumor cells (multiple cancer types)	Shift from conventional (CD49b^+^) to tissue resident (CD49a^+^), with increase in inhibitory receptors and decrease in activating receptors.	[118,119]
	Breast cancer cells	Interactions result in NK reprogramming to a tumor-promotional phenotype.	[120]
	Tumor cells (multiple cancer types)	Transfer of cell surface proteins by trogocytosis; transfer of PD-1 inhibitory receptor or NKG2D suppresses NK function, while TYRO3 enhances function.	[121–123]
	CAFs	Inhibit NK tumor infiltration and cytotoxicity through collagen deposition, and protect cancer cells from NK attack by acting as a decoy.	[124,125]
	Hepatic stellate cells	Inhibit NK cell elimination.	[126]
Multiple myeloid cell types (DCs, MDSCs and macrophages)	Tumor cells (multiple cancer types)	Cancer cell transfer of extracellular vesicles containing microRNAs alters activities of myeloid cells to be immunosuppressive.	[127–130]
Multiple immune cell types	Astrocytes and glial cells	Promote an immunosuppressive microenvironment.	[131,132]
	Mesenchymal stem cells	Can be immune-promotional or immune-suppressive.	[133,134]

CAF, cancer-associated fibroblast; DC, dendritic cell; MDSC, myeloid-derived suppressor cell; NET, neutrophil extracellular trap; NK, natural killer.

### T cell differentiation and cell fate

T cells are adaptive immune cells that eliminate diseased cells, whether infected or cancerous [135], and while cytotoxic CD8^+^ T cells can be potent tumor-killers, other types of T cells, such as CD4^+^ helper T cells, coordinate immune responses and can at times promote immune evasion. Further, T cells that were once cytotoxic can become over-activated to the point of exhaustion and lose their cytotoxic capabilities [136], which forms the basis for T-cell-targeted immune checkpoint inhibitors such as anti-PD1/PDL1 and anti-CTLA-4 therapies. Tumor cells significantly alter T cell differentiation and fate, contributing to mutual plasticity in the TiME.

Re-instatement of developmental programs in tumor cells, as is observed in triple negative breast cancer (TNBC) and many other cancers, not only alters the plasticity of the tumor cells themselves, but also alters the plasticity of the immune microenvironment [113,137–141]. For example, re/over-expression of the transcriptional co-factor EYA3, which belongs to the family of Eyes absent (EYA) developmental regulators, promotes TNBC aggressiveness by suppressing CD8^+^ T cells via increasing PDL1 expression on the TNBC cells, through a mechanism that is dependent on regulation of MYC by EYA3 [113]. This restoration of a developmental function for EYA proteins thus induces plasticity not only in the tumor cells, but also influences the TiME [113,142].

Tumors develop hypoxic regions as they grow, and this lack of oxygen can impact the activity and function of T cells in the TiME [114]. For example, tumor hypoxia induces a MYC-regulated mitochondrial defect within T cells that promotes their exhaustion [114]. Further, targeting miR-24 or the MYC/FGF11–MFN1 signaling axis could reverse the exhausted T cell fate or prevent it from happening, which may improve the efficacy of T-cell-targeted immunotherapies [114].

Mitochondrial dysfunction also occurs within tumor infiltrating T cells, but this defect does not always arise through signaling or conditions generated by the tumor; instead, it can arise through direct transfer of material from tumor cells themselves [115]. Mitochondria can be transferred between cells within the TiME through extracellular vesicles (EVs) or tunneling nanotubes. In one study, cancer cells transferred mitochondria with mutated mitochondrial DNA to T cells, and the mitochondria took up residence rather than being degraded through mitophagy, as mitophagy-inhibitory molecules were transferred along with the mitochondria [115]. Such mitochondrial DNA mutations led to metabolic abnormalities, defects in effector functions and memory formation, and senescence in T cells, altogether diminishing the effects of immunotherapy. These studies underscore the multifaceted ways in which tumor cells actively contribute to T cell reprogramming.

### Dynamic changes in NK cells

NK cells both eliminate cancer cells and promote the development of a replete immune response through the secretion of cytokines that boost antigen presenting cells and T cells. NK cells adopt heterogenous phenotypes in different tissue types and in response to various stimuli, broadly categorized as mature/cytotoxic, or less mature/cytokine-producing, marked by expression of CD56 and CD16 in humans [143,144] or CD11b and CD27 in mice [145,146]. Recently, single-cell sequencing analysis has led to further refinement of NK cell phenotypes and revealed that NK cell subset distribution varies widely by tumor type [147]. Similar findings were observed in another large scale single-cell RNA sequencing analysis of patient samples across 24 cancer types, which demonstrated that NK cell heterogeneity was specific to tumor type and that tumor-infiltrating NK cells had reduced cytotoxic features compared to blood-derived NK cells [148], suggesting that NK cells shift their phenotype upon tumor entry. In support of this finding, Kaede photoconvertible mice were used to dynamically label tumor-infiltrating lymphocytes, and it was observed that NK cells entering the tumor shifted from CD49b^+^ conventional NK cells to CD49a^+^ tissue-resident NK cells, accompanied by an upregulation of inhibitory receptors and reduced expression of cytokines associated with DC recruitment [118].

Tumor cells can also influence NK cell behavior in metastasis. For example, TNBC cells expressing EYA3 can inhibit the recruitment/presence of cytotoxic NK cells at the pre-metastatic niche [138], and tumor cells can promote NK cell dysfunction in the metastatic niche to allow for outgrowth [149]. Beyond reducing NK cell cytotoxic potential and infiltration, NK-cell–tumor crosstalk can push NK cells to a pro-tumor state, and tumor-educated NK cells enhance metastatic outgrowth [120]. Thus, through multiple mechanisms, tumor cells alter NK cell phenotype and activity to promote tumor progression.

Tumor cell supernatant alone can be sufficient to induce changes in the expression of NK cell receptors, shifting them from the expression of more activating receptors to more inhibitory receptors, suggesting a role for tumor cell-secreted soluble factors in suppressing NK cell activity [119,138]. However, direct contact with tumor cells can also induce changes to NK cell receptor cell surface levels through a process called trogocytosis, whereby immunological synapses that form between NK cells and target cells allow for the exchange of plasma membrane and associated proteins [150,151]. Trogocytosis can result in the suppression of NK cell-mediated anti-tumor immunity through acquisition of inhibitory receptors [121], or transfer of activating ligands that promote recognition and fratricide by other NK cells [122]. Trogocytic acquisition of tumor antigen can limit the therapeutic efficacy of an immunotherapy in which NK cells are manipulated to recognize a specific antigen on tumor cells termed chimeric antigen receptor (CAR)-NK, but more advanced dual-CAR systems or co-treatment strategies can prevent this issue [152,153]. Alternatively, trogocytosis of TYRO3 receptor tyrosine kinase from tumor cells can enhance NK cell cytotoxicity and activation [123], and trogocytosis of CCR7 promotes NK cell migratory capability [154]. Altogether, through indirect and direct interactions, tumor cells provoke dynamic changes in tumor-infiltrating NK cells.

### Myeloid diversity and reprogramming by tumor cells

Myeloid cell diversity and plasticity arise from many factors including cellular ontogeny [155], niches the cells develop within or are recruited to [156] and, in a tumor context, how myeloid cells are spatially organized [157] and which signals they are receiving from tumor cells, the latter of which will be the focus of this section. Throughout tumor progression, tumor cell signaling can contribute to the reprogramming of myeloid cells such that they become either suppressed and/or actively pro-tumorigenic [69,70]. This reprogramming can occur through secreted factors or direct cell–cell contact shared between cancer and immune cells, which has been reviewed extensively elsewhere [158–162]. Here, we highlight novel examples of how tumor cells reprogram or recruit myeloid cells through epigenetic regulation, metabolic reprogramming and release of EVs to support tumor growth.

Epigenetic regulation modulates gene expression without changing the sequence of DNA. This regulation can occur through mechanisms including histone modification, DNA methylation or through the activity of non-coding RNAs [163]. Epigenetic changes within cancer cells can promote reprogramming and recruitment of myeloid cells, sometimes by also inducing epigenetic changes within the myeloid cells themselves [163]. CECR2, an acetyl-lysine reader, is a top upregulated epigenetic regulator in metastatic breast cancer that interacts with RELA and is recruited to NFκB target genes that promote immunosuppressive macrophage polarization (Box 2) [94]. In pancreatic cancer, the H3K36 trimethyltransferase SETD2 regulates neutrophil recruitment [109]. Tumor cell intrinsic SETD2-H3K36me3 loss increases PI3K–AKT signaling, leading to robust expression of CXCL1 and GM-CSF, that together recruit neutrophils and reprogram them to be immunosuppressive [109]. There is also abundant evidence for epigenetic regulators in cancer cells promoting the recruitment of MDSCs to the TiME [103–106]. For example, in colorectal cancer, the m^6^A methyltransferase METTL3 promotes transcription factor m^6^A-BHLHE41 to increase CXCL1 transcription, which when secreted, recruits immunosuppressive MDSCs to the tumor [104].

Cancer cells undergo metabolic reprogramming to support their high energy demands for rapid growth, and their unique metabolic activity can promote pro-tumor reprogramming in myeloid cells [164]. Lactate accumulates in the TiME as a result of excessive aerobic glycolysis (Box 2) in tumor cells, which supports immunosuppressive macrophage polarization [98], increased release of pro-tumor factors by neutrophils, as well as NETs, which feedback to induce more lactate production [107,108] and limit the ability of DCs to present antigen to T cells [111]. Cancer cells also alter the Krebs cycle to support their rapid growth [164]. In cholangiocarcinoma, a key enzyme in the Krebs cycle, PDHA1, is succinylated, leading to α-ketoglutaric acid accumulation in the TiME [93]. The α-ketoglutaric acid accumulation ultimately promotes MAPK signaling within the macrophages and inhibits their ability to present antigen [93]. Altered lipid metabolism is another way cancer cells meet their energetic needs [164]. Cancer cells up-regulate *de novo* lipogenesis as well as lipid uptake, and this resulting increase in the fatty acid pool can impact signaling to immune cells [28]. For example, the abundance of fatty acids in tumors promotes their uptake by macrophages, which results in an accumulation of cytoplasmic lipid droplets [95]. Metabolism of the lipid droplets within macrophages can induce pro-tumor macrophage reprogramming through multiple pathways [96,97]. A similar phenomenon occurs in DCs, in which accumulation of extracellular lipids, such as tumor-derived lipids, inhibits their ability to present antigen and stimulate T cells [112,165].

A unique way in which tumor cells communicate through secreted factors is via the release of EVs, particles of membrane-encapsulated cellular material (e.g., DNA, RNA, protein, metabolites) that range in size from 40−100 nm in the case of exosomes, and up to 800−5,000 nm in the case of apoptotic bodies [166]. Tumor-derived exosomes, or TEX, can send short-range or long-range signals to immune cells, as they can travel through the circulation, by transferring their contents into the recipient cell, or by signaling through receptor–ligand interactions [129]. MicroRNAs (miRNAs) are amongst the most abundant cargo in TEX and can change gene expression when transferred to the recipient myeloid cell. Some examples include: TEX with miR-212-3p released from pancreatic cancer cells can impair antigen presentation by DCs [127]; TEX containing miR-29a and miR-92a from glioma bolster the expansion of MDSCs [128]; and TEX containing miR-301a-3p from hypoxic pancreatic cells polarize macrophages to a pro-tumor phenotype [129,130]. Finally, cancer cells can also signal to myeloid cells through apoptotic debris: another form of EV. Sporadic apoptosis of colorectal cancer cells recruits neutrophils through IL-8 signaling and induces a pro-tumor phenotype within neutrophils when they take-up apoptotic debris [99]. Together, these studies illustrate how tumor cells can contribute to reprogramming of diverse myeloid populations through a variety of mechanisms to promote cancer progression.

## Additional modulators of tumor–immune crosstalk

Tumor–immune interactions in the TiME are not only impacted by the activities of tumor and immune cells themselves, but also by plasticity that results from crosstalk between additional diverse cell populations (summarized in Table 1 and Fig 1).

### Stromal cell influence on immune cells in the TiME

Tumor-infiltrating immune cells encounter multiple cell types in the TiME, including fibroblasts, endothelial cells, mesenchymal stem cells, nerve cells, adipocytes and pericytes [167], and crosstalk between stromal and immune cells significantly influences anti-tumor immune responses. Cancer-associated fibroblasts (CAFs) are a prominent component of the TiME and contribute to tumor growth and progression [168]. CAFs are diverse in origin, thought to be derived from fibroblasts, adipocytes, pericytes, endothelial cells and mesenchymal cells, and display extraordinary heterogeneity and phenotypic plasticity. CAF activity in the TiME directly inhibits NK cell infiltration and cytotoxic killing in breast cancer tumors through collagen deposition [124], and direct physical interaction between CAFs and NK cells protects cancer cells from NK cell-mediated elimination by acting as a decoy target for killing [125]. CAF-secreted factors can also inhibit NK cells indirectly by polarizing macrophages and monocytes towards a suppressive phenotype [101,102]. In addition to effects on NK cells and macrophages/monocytes, CAFs influence the activity of T cells [169], DCs [126] and endothelial cells [170]. Interestingly, crosstalk between antigen-presenting CAFs and macrophages in gastric cancer supports anti-tumor T cell responses [116], suggesting that CAFs may promote or inhibit anti-tumor immune responses depending on their crosstalk with other cells in the TiME.

Beyond CAFs, diverse stromal cell types influence immune–cancer crosstalk. In the TiME, endothelial cells can undergo an endothelial–mesenchymal transition and adopt fibroblast-like features [171]. Endothelial cells that have undergone this process support polarization of tumor-associated macrophages and resistance to radiation therapy [100]. Mesenchymal stem cells exhibit extraordinary phenotypic plasticity, and can have immune suppressive or promotional effects depending on context [133,134]. Interestingly, mesenchymal stem cells are known for their advanced ability to migrate into tumors, which has made them an attractive vehicle for the delivery of anti-cancer compounds, and multiple groups have engineered biologic therapies using mesenchymal stem cells [172].

Stromal effects can also be organ-specific, depending on crosstalk with specialized tissue-resident cell types. For example, in the brain, astrocytes and glial cells promote an immunosuppressive microenvironment that supports brain tumor growth [131,132], while in the bone, osteoclasts protect multiple myeloma cells from T cell attack [117], and in the liver, hepatic stellate cells inhibit NK cells to enable breast cancer metastatic outgrowth [126]. Altogether, diverse interactions between heterogeneous stromal cells and phenotypically plastic immune cells have profound effects on tumor growth and progression.

### Tumor crosstalk with hematopoietic niches

In addition to crosstalk occurring between cancer cells and local cells within the TiME, cancer cells can crosstalk with distant tumor-relevant niches, such as hematopoietic niches within the bone marrow. Aside from yolk sac-derived immune cells, most immune cells originate from hematopoietic stem cells in the bone marrow, and then mature in a stepwise fashion to the lineage-committed immune cell types discussed in this Essay. Interestingly, recently published research has demonstrated that immune cell progenitors within the bone marrow can distantly communicate with tumor cells. For example, in non-small cell lung cancer (NSCLC), immunosuppressive polarization of myeloid cells in response to tumor burden largely occurs in the bone marrow [173]. Eight cytokines secreted from NSCLC cells work collaboratively to induce IL-4 production from bone marrow basophils and eosinophils. IL-4 then signals through IL-4Rα on granulocyte-monocyte progenitor cells, which transcriptionally programs them to become tumor-promoting myeloid cells as they mature [173]. A clinical trial was initiated from this study that combined the IL-4Rα blocking antibody, dupilumab, with PD-1/PD-L1 checkpoint blockade in patients with NSCLC who had progressed on checkpoint blockade alone [173]. This combination resulted in positive clinical outcomes including reducing circulating monocytes and expanding tumor-infiltrating CD8^+^ T cells, thus providing an additional avenue for overcoming therapy resistance [173].

Others have characterized breast and lung cancer-induced changes to hematopoietic niches pre- and post-tumor removal [174]. For example, remote tumors can reprogram the bone marrow microenvironment by promoting osteoprogenitor cell–granulocyte-monocyte progenitor cell interactions through EV signaling, leading to overproduction of immunosuppressive myeloid cells. Intriguingly, this effect persists after tumor removal, which may contribute to therapy resistance. Tumor–hematopoietic niche crosstalk is not only relevant to the primary tumor, but also to metastatic spread. For example, mammary tumors can accelerate neutrophil lineage commitment from bone marrow progenitors, thereby polarizing neutrophils to an immunosuppressive phenotype that then enhances metastasis [175]. These representative studies highlight the critical role of tumor–hematopoietic niche crosstalk in shaping immune populations, promoting tumor growth and metastasis as well as therapy resistance, and further underscore the need for new therapeutic strategies that target this crosstalk.

### Tumor cell innervation

Tumor innervation and co-opting of neural circuits by cancer cells have recently emerged as processes underlying regulation of six of the ten current ‘hallmarks of cancer’ [176]. The peripheral nervous system comprises autonomic and sensory nerves that help maintain homeostatic control of the body at the molecular, cellular and organ level. Thus, when equating solid tumors to ‘aberrant neo-organs’, it is not surprising that innervation is also critical to pro-survival processes within them [177]. The autonomic nervous system, which regulates involuntary physiological functions, is divided into the sympathetic and parasympathetic nervous systems, both which have post-ganglionic cell bodies that sit in proximity to the organs they innervate. The post-ganglionic nerves respond to the microenvironments they reside within, including the TiME [177].

Innervation of the TiME was first observed over a century ago [178], and since then, detection of increased nerve density has been reported across multiple tumor types including pancreatic, prostate, breast, colorectal, lung, and head and neck, amongst others [179]. Only more recently have we begun to mechanistically understand how nerves regulate tumor growth. Sympathetic nerves support tumor growth, while parasympathetic nerves and sensory nerves can have divergent effects depending on the tumor type [179]. Nerves can also alter tumor cell plasticity by acting on cancer cells directly in brain tumors and breast cancer brain metastases [180]. Functional synapses can be formed between neurons and glioma cells, while perisynaptic interactions occur between neurons and breast cancer cells within the brain, or with glioma cells. In the perisynaptic interaction, two neurons interact, while the cancer cell is positioned perpendicular to the synapse to also receive a depolarizing signal via glutamate through NMDA receptors [180]. NMDA receptor signaling in cancer cells can promote proliferation, migration and invasion [181]. Furthermore, sensory nerves play a role in breast cancer metastasis. While it was previously shown that nerves can promote metastasis through a process called perineural invasion [182], more recent work demonstrates that neuronal substance-P (SP) can also drive metastasis in breast cancer through a mechanism that does not require physical contact between nerves and cancer cells [183]. Instead, breast cancer cells induce spontaneous calcium activity in sensory neurons, which leads them to release SP, feeding back on the tumor cells to promote a pro-metastatic gene expression program. This pro-metastatic program arises through activation of TLR7 by single-stranded RNA released during cell death induced by SP acting on tumor cells with high expression of TAKR1. Excitingly, aprepitant, an anti-nausea drug, inhibits this neuro–cancer axis and inhibits tumor growth and metastasis in multiple models [183]. These discoveries position neural signaling as a critical and previously underappreciated driver of several cancer types.

### Targeting plasticity as a future means of inhibiting tumor progression

Current therapeutic strategies have largely focused on inhibiting proteins and signaling pathways that allow tumors to proliferate unchecked and evade immune responses. However, tumor–immune plasticity in response to these interventions presents an immense challenge. Thus, therapeutic strategies that specifically target this plasticity are vital for successful long-term responses.

One potential avenue involves preventing EMT induction. TGFβ is a strong inducer of EMT, and also exerts immunosuppressive effects, making it an attractive target for therapy, especially in combination with immune checkpoint inhibitors. Multiple strategies to inhibit TGFβ have been developed, including small-molecule inhibitors, monoclonal antibodies, ligand traps and anti-sense oligos [184]. Therapeutic targeting of TGFβ in combination with immune checkpoint blockade has shown promising results in preclinical models [185,186], and can even promote the transition of the tumor immune phenotype from immune-desert to immune-inflamed [2]. However, results from clinical trials have been inconsistent and underwhelming, with TGFβ inhibition not showing much added benefit [185]. The combination therapy strategy may be more beneficial in certain tumor types [185], or in the context of advanced tumors [187], given that TGFβ levels increase with disease progression and correlate with response [188]. Agents that target other regulators of EMT are also under investigation in preclinical and clinical studies, including inhibitors of the EMT-promoting receptor tyrosine kinase AXL, either alone or in combination with immunotherapy [189], and an antibody against netrin-1, which inhibited EMT in endometrial cancer [190].

One caveat of inhibiting EMT induction is that it may cause the reverse phenomenon, mesenchymal–epithelial transition, a process that contributes critically to metastasis [191]. Because multiple cell states contribute to tumor progression, a preferred approach to inhibiting tumor progression may be to target cancer cell plasticity to prevent phenotype switching, rather than targeting the process of EMT per se. Epigenetic regulators facilitate phenotype switching [192], and epigenetic targeting drugs have shown promise in preclinical and early clinical studies for modulating EMT and tumor plasticity [192,193]. Such inhibitors can destabilize the gene expression programs that drive EMT and increase sensitivity to conventional therapies [194–196]. Since epigenetic changes within tumor cells can also generate an immunosuppressive microenvironment, inhibiting epigenetic regulators may promote a more permissive environment for immune cells [94,109]. Another strategy is to eliminate cells that have already undergone EMT. Along these lines, in patients with lung cancer who have tumors that have become resistant to EGFR inhibitors, EMT induction results in increased expression of the cell surface protein CD70, opening up the possibility to use new interventions involving CAR-NK or antibody–drug conjugates that specifically guide therapeutic agents to CD70-expressing cells in order to eliminate drug-resistant cells that have undergone EMT [197]. In cutaneous squamous cell carcinoma, immune checkpoint expression also changes throughout tumor progression, rendering tumors differentially sensitive to various immune checkpoint inhibitors based on the epithelial–mesenchymal make-up of the tumor [198]. These data suggest that treatment may need to be tailored to an individual based on target gene expression.

Moreover, targeting additional cell types in the TiME, ones that are plastic themselves or that promote cancer cell plasticity, could be an advantageous approach to inhibiting tumor progression. Myeloid cells exhibit incredible plasticity and can be relatively easily manipulated towards a pro-tumor or anti-tumor phenotype, and multiple therapeutic strategies have been proposed to modulate myeloid cell behavior in tumors [199]. Fibroblast plasticity promotes dynamic changes in the microenvironment to favor tumor cell survival, and strategies to alter CAF differentiation and signaling have been proposed as a means to inhibit this tumor-promotional crosstalk [200]. Tumor innervation often promotes tumor progression, and inhibiting tumor–nerve interactions through surgical or chemical methods have been effective at inhibiting or slowing the occurrence and spread of prostate cancer and pancreatic ductal adenocarcinoma, as well as the development of gastric cancer and non-melanoma skin cancers [201–205]. Mutual plasticity between cancer cells, immune cells and stromal cells critically contributes to tumor progression and adaptability under therapeutic pressure, and targeting this plasticity is essential for long-term benefit in the clinic.

## Next steps in this rapidly evolving field

The mutual plasticity of cancer and immune cells in the TiME poses a major challenge to both scientific study and therapeutic advancements. Diverse phenotypes that shift over time are difficult to capture in model systems (Box 1), but advanced technologies have expanded our ability to study these adaptive changes (Box 3). Such technologies includes spatial imaging, which can capture the spatial distribution of cells with specific gene and protein expression in a heterogeneous tumor, information that is usually lost in sequencing and flow cytometry data. This spatial understanding is critical for the study of cancer–immune crosstalk and the development of novel therapeutic strategies, with data supporting a role for specific spatial architectures in informing the success of anti-tumor immune responses and immunotherapy [157,206,207]. Microfluidics and organ-on-chip platforms have also enabled a deeper investigation of cancer cell plasticity and crosstalk in different organs, as well as enabling high throughput study of potential drug treatments in an *in vitro* model system that better recapitulates the TiME [208–210]. Crosstalk between cancer, immune and stromal cells mediates complex feedback loops that induce plasticity across these cell types, and advanced mathematical and computational models have emerged as invaluable tools to understand the innumerable phenotypes that may develop over the course of tumor cell state transitions [211–214].

**Box 3 Advanced technologies for studying plasticity and the TiME.**

**Spatial technologies and niche labeling**

Spatial technologies enable the mapping and quantification of cancer and immune cells types and interactions in the tumor immune microenvironment (TiME). Multiplexed immunofluorescence uses fluorescently labeled antibodies that allow for visualization of up to dozens of markers [215]. Spatial transcriptomics [216] and spatial proteomics [217] enable the measurement of RNA or protein expression, respectively, across tissue sections at single-cell resolution. Multiplexed ion beam imaging uses metal-tagged antibodies in conjunction with mass spectrometry to image thirty or more proteins simultaneously with subcellular localization [218].

Advanced labeling techniques have enabled higher resolution tracing of interacting cells in the TiME. In the Cherry-niche system, metastatic cells secrete a fluorescent protein that labels neighboring cells, allowing for spatial resolution of interactions between cells residing in the immediate TiME [219]. Additional systems have been developed to label cells that interact with one another [217,220], with the ability to mark encounters between cancer cells and immune cells or between immune cells themselves, allowing for advanced temporal and spatial mapping of interactions in the metastatic niche.

**Organ-on-chip and microfluidics *in vitro* models**

When cancer cells metastasize to distant sites, their characteristics change as they travel in the bloodstream and change again when they interact with a new microenvironment upon arrival at the secondary organ. The plasticity involved in cancer cell trafficking through the bloodstream during metastasis, and cancer–immune crosstalk at secondary organ niches, can be difficult to model without utilizing live animal models. Microfluidics platforms have enabled enhanced characterization and study of circulating tumor cells that travel in the bloodstream [209], and modeling of difficult microenvironments such as the bone marrow [210]. Furthermore, integration of microfluidics platforms along with 3D model systems and tissue engineering has led to the development of tumor/organ-on-a-chip devices that can recapitulate the TiME of different organ environments [221]. While these systems allow for high throughput studies *in vitro*, caveats include the lack of integration of signals from other complex systems such as immune or endocrine inputs, and differences in manufacturing of chips that create critical differences in studies between labs [221].

**Computational models**

The complex interplay between cells in the TiME can be difficult to study utilizing *in vitro* and *in vivo* model systems, which either provide only a snapshot of cancer cells and immune cells, or do not fully mimic the human condition. The potential for heterogeneous cancer cells to adopt different phenotypes over time introduces further complexity into these studies, such as is observed with EMT, where cells can exist in a spectrum of epithelial–mesenchymal transition (EMT) hybrid states. Innovative mathematical models have enabled the complex analysis of this plasticity, providing a framework to understand the extensive phenotypes that may arise during EMT (and mesenchymal–epithelial transition), the reversibility of this process and the effect of crosstalk between cancer cells on EMT induction in neighboring cells [214]. Using documented cellular interactions, computational models have been developed to predict the outcomes of interactions between various cell states and to further understand how plasticity influences immune–cancer crosstalk [213]. By further integrating specific patient biological information, these tools can be utilized to predict clinical outcomes and potential drug combinations for use in the clinic [211,212].

Cancer is a complex disease to study and treat, in part due to the intricate signaling interplay between cancer cells, immune cells and other local and distant members of the TiME. The phenotypic plasticity induced by these interactions creates a highly heterogenous microenvironment that can enable tumors to evade anti-tumor immune responses and become resistant to therapeutic intervention. However, as our understanding of this mutual plasticity improves, so does our ability to harness these tumor–TiME interactions for therapeutic gain. Continued exploration of this plasticity through use of the model systems (Box 1) and advanced techniques (Box 3) outlined here and beyond, will pave the way for more precise and durable therapeutic strategies.

## Abbreviations

CAFs: cancer-associated fibroblasts
EMT: epithelial-to-mesenchymal transition
METs: macrophage extracellular traps
NETs: neutrophil extracellular traps
NK: natural killer
NSCLC: non-small cell lung cancer
TiME: tumor immune microenvironment
TNBC: triple negative breast cancer
